# The multikinase inhibitor midostaurin (PKC412A) lacks activity in metastatic melanoma: a phase IIA clinical and biologic study

**DOI:** 10.1038/sj.bjc.6603331

**Published:** 2006-09-12

**Authors:** M J Millward, C House, D Bowtell, L Webster, I N Olver, M Gore, M Copeman, K Lynch, A Yap, Y Wang, P S Cohen, J Zalcberg

**Affiliations:** 1 Peter MacCallum Cancer Institute, Melbourne, Victoria, Australia; 2 Royal Adelaide Hospital, Adelaide, South Australia, Australia; 3 Royal Marsden Hospital, London, UK; 4 Novartis Pharmaceuticals Australia Pty Ltd, North Ryde, New South Wales, Australia; 5 Novartis Pharmaceuticals Inc., East Hanover, NJ, USA

**Keywords:** protein kinase C, melanoma, midostaurin

## Abstract

Midostaurin (PKC412A), *N*-benzoyl-staurosporine, potently inhibits protein kinase C alpha (PKC*α*), VEGFR2, KIT, PDGFR and FLT3 tyrosine kinases. In mice, midostaurin slows growth and delays lung metastasis of melanoma cell lines. We aimed to test midostaurin's safety, efficacy and biologic activity in a Phase IIA clinical trial in patients with metastatic melanoma. Seventeen patients with advanced metastatic melanoma received midostaurin 75 mg p.o. t.i.d., unless toxicity or disease progression supervened. Patient safety was assessed weekly, and tumour response was assessed clinically or by CT. Tumour biopsies and plasma samples obtained at entry and after 4 weeks were analysed for midostaurin concentration, PKC activity and multidrug resistance. No tumour responses were seen. Two (12%) patients had stable disease for 50 and 85 days, with minor response in one. The median overall survival was 43 days. Seven (41%) discontinued treatment with potential toxicity, including nausea, vomiting, diarrhoea and/or fatigue. One patient had >50% reduction in PKC activity. Tumour biopsies showed two PKC isoforms relatively insensitive to midostaurin, out of three patients tested. No modulation of multidrug resistance was demonstrated. At this dose schedule, midostaurin did not show clinical or biologic activity against metastatic melanoma. This negative trial reinforces the importance of correlating biologic and clinical responses in early clinical trials of targeted therapies.

Midostaurin (PKC412A, CGP41251), *N*-benzoyl-staurosporine, is a potent inhibitor of calcium- or diacylglycerol-dependent isoforms of protein kinase C (PKC*α*, PKC*β*I, PKC*β*II and PKC*γ*), and VEGF-R2, PDGFR, KIT and Flt-3 kinases (Fabbro *et al*, 2000; Weisberg *et al*, 2002). Although midostaurin is a competitive inhibitor of ATP binding to PKC*α*, midostaurin is most active in inhibiting particulate (active) PKC enzymes from tumour samples (Killion *et al*, 1995; Ikegami *et al*, 1996).

*In vitro*, midostaurin slows tumour cell growth, in G2/M phase, inducing polyploidy, apoptosis and radiosensitivity, with inhibition of the PI3K/Akt pathway (Fabbro *et al*, 2000; Tenzer *et al*, 2001). *In vivo* midostaurin inhibits growth of several murine tumours, potentiates paclitaxel, doxorubicin and radiation, and may affect multidrug resistance (Utz *et al*, 1994; Fabbro *et al*, 2000). Angiogenesis is also inhibited by midostaurin (Ozaki *et al*, 2000).

The incurability of metastatic melanoma indicates a need for new therapy, preferably targeted against molecular changes associated with tumorigenicity (Brown and Kirkwood, 2003). Protein kinase C alpha activity is elevated in some melanoma cells and is a potential target (Selzer *et al*, 2002). Midostaurin inhibits PKC*α* activity in melanoma cells, and delays their lung metastasis in mice (Yoshikawa *et al*, 2003). So, midostaurin might be useful clinically in patients with metastatic melanoma.

In a Phase I trial, 32 patients with advanced cancer received oral midostaurin at 12.5–300 mg day^−1^ (Propper *et al*, 2001). Between two patients with cholangiocarcinoma, one had stable disease and one a partial response for 4 months on midostaurin. No other responses were seen. Frequent but mild toxicities were nausea, vomiting, fatigue and diarrhoea. MTD was not reached, but at 225–300 mg day^−1^, 15 out of 16 patients had nausea/vomiting (grade 3 in 3); and six out of 16 had grade 2 diarrhoea. There was minimal myelosuppression.

Midostaurin dose and AUC (0–24 h) showed linear correlation, but with marked inter-patient variability. Estimated median elimination *t*_1/2_ was 1.6 days (range: 0.9–4.0 days). Several active metabolites, including one (CGP52421 e2) with a median *t*_1/2_ of 36 days, were also detected (Figure 1).

The objectives of this Phase IIa trial of midostaurin in patients with advanced metastatic melanoma were to evaluate midostaurin's effects on measurable disease, to further define its toxicity, and to investigate drug concentrations and biological activity in tumour biopsies and plasma.

## PATIENTS AND METHODS

### Eligibility

Patients with stage IV metastatic melanoma that had progressed despite any previous therapy were eligible if they were 18 years of age or older, had an ECOG performance status of 0–2, had adequate haematologic, renal and hepatic function, had bidimensionally measurable disease, and were able to provide informed consent. There was no limit on the amount of prior therapy, but no prior chemotherapy or immunotherapy was permitted in the 4 weeks before study entry. Patients with CNS metastases were eligible. At least the first 14 patients entered were also required to have at least two lesions suitable for excision biopsy (skin, subcutaneous metastases). Written informed consent was obtained from all patients and the protocol was approved by the Human Research Ethics Committees of the participating institutions.

### Treatment

All patients were treated with midostaurin 75 mg (6% w w^−1^ in Gelucire 44 out of 14) given orally three times a day (225 mg day^−1^) continuously until disease progression or unacceptable toxicity. This fixed dose schedule was calculated from the Phase I study, in which higher single daily doses were associated with more GIT toxicity. However, if grade 3 toxicity considered related to midostaurin occurred, treatment was withheld for up to 7 days then continued at 75 mg twice daily (150 mg day^−1^). Patients were instructed to take midostaurin with food while sitting upright, to reduce nausea.

Patients were reviewed for safety and efficacy of midostaurin weekly for the first month, and then on a monthly basis, or more frequently where symptoms warranted. Disease sites were re-evaluated by clinical examination monthly or CT every 2 months, or more frequently if disease progression was suspected.

### Assays for biologic activity

**Figure 1:**
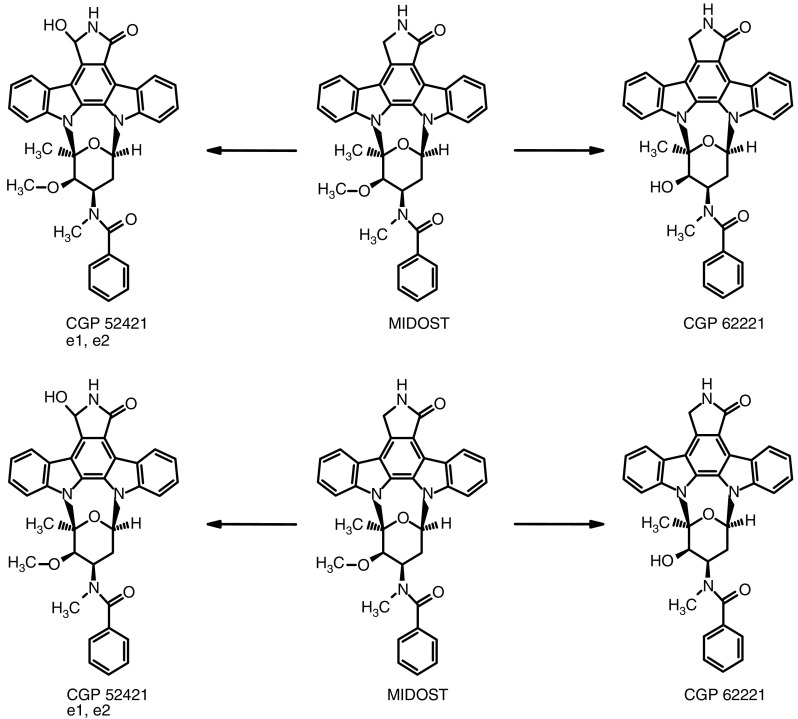
Chemical structures of MIDOSTAURIN and its *O*-desmethyl metabolite CGP62221 (O-D-MIDOSTAURIN) and 7-hydroxy metabolite CGP52421 (7-OH-MIDOSTAURIN), the later has two epimers, e1 and e2.

To determine any biologic activity of midostaurin, patients had tumour biopsies and plasma taken for analyses in the week prior to starting midostaurin and again, where practical, on day 28. Tumour biopsies were immediately frozen in liquid nitrogen and stored at −70°C until analysis. Heparinised blood (10 ml) was collected predose and at day 28. Following centrifugation, plasma was stored at −70°C until assay for MDR-reversing ability, and for concentrations of midostaurin and its two main metabolites CGP62221 and CGP52421 e2 (Figure 1).

#### Preparation of cytosolic and particulate fractions from tumour biopsies

Tumours were powdered in liquid nitrogen and homogenised on ice using an Ultra-Turrax in a buffer containing 20 mM HEPES, pH 7.4, 2 mM EDTA, 25 mM *β*-glycerophosphate, 0.2 mM DTT, 10 *μ*g ml^−1^ leupeptin, 10 *μ*g ml^−1^ aprotinin, 1 *μ*g ml^−1^ pepstatin, 1 *μ*M sodium orthovanadate and 0.5 mM PMSF. Extracts were then sonicated in an ice cold sonicating bath for 5 min before centrifugation at 1000 **g** for 5 min to remove cellular debris. Supernatants were centrifuged at 50 000 **g** for 15 min to separate cytosolic (supernatant) and particulate (pellet) fractions. The supernatant was kept as ‘cytosolic’ fraction. The pellet was further extracted by resuspension in the original lysis buffer including 1% NP-40. Following sonication for 5 min on ice, the extract was centrifuged at 50 000 **g** for 15 min. The supernatant from this spin (which derived from the original pellet) was kept as the ‘particulate’ fraction. All steps were performed at 4°C or on ice.

#### Protein measurement

The protein concentrations of the cytosolic and particulate fractions were measured after TCA precipitation using the Dc protein assay kit (Bio-Rad).

#### PKC activity measurements

Intratumoral total PKC activity was measured in cytosolic and particulate fractions using protamine sulphate as the substrate, following the method of a validated assay (Budworth and Gescher, 1995). The PKC assay was performed with 4–5 *μ*l of the cytosol or particulate extract (2–50 *μ*g protein) in a total reaction volume of 200 *μ*l, including 200 *μ*g ml^−1^ protamine sulphate, 150 *μ*M (*γ*-^32^P)ATP (200 c.p.m. pmol^−1^) in a buffer containing 20 mM Tris-HCl, pH 7.4, 10 mM magnesium acetate and 0.5 mM EGTA. The reaction was terminated after 6–7 min (30°C) by spotting 35 *μ*l of the mix onto P81 phosphocellulose paper, followed by washing in 75 mM phosphoric acid. Bound, phosphorylated protamine sulphate was quantitated by scintillation counting of the phosphocellulose papers. Protein kinase C activity was determined by phosphate incorporation into protamine sulphate and expressed as picomolar of phosphate transferred min mg^−1^ protein. Measurements were performed in triplicate. Each patient's pretreatment and day 28 biopsies were processed together and analysed in the same assay run. Up to six patients’ samples could be analysed in one day to minimise interassay variability. Initial experiments on melanoma biopsies from nontrial patients showed addition of 10 *μ*M midostaurin to melanoma biopsies inhibited phosphorylation.

#### Western blotting for PKC isoform abundance

For three patients, 2 *μ*g of cytosol and particulate proteins from baseline and day 28 biopsies were resolved on 15% acrylamide SDS-PAGE gels, transferred onto PVDF membrane and probed with antibodies to PKC isoforms (Santa Cruz).

#### Drug concentrations in plasma and tumour tissue

Plasma concentrations of midostaurin and its two main metabolites CGP 62221 and CGP52421 e2 were determined using an HPLC method with fluorescence detection as reported previously (Van Gijn *et al*, 1995; Propper *et al*, 2001). Homogenisation of the tumour tissue by freeze-fractionating proved impossible. Therefore, the tumour tissue was frozen and cut into as small as possible pieces with a scalpel. The pieces were weighed, added to 1 ml of water, vortexed and thereafter treated like plasma. The assay for plasma samples was validated with acceptable calibration curves, within-study variability and total recovery. The limit of quantitation (LOQ) in these analyses (defined as the concentration of the lowest QC sample with a mean recovery of 80–120% and a CV ⩽20%) was 36 nmol l^−1^ for MIDOSTAURIN, 61 nmol l^−1^ for CGP 62221, and 265 nmol l^−1^ for CGP52421 e2. Due to limited availability of tumour tissue, the within-study validation for tumour assay was not performed. The tissue assay showed good recovery for PKC412, 95.8±6.2%, and for CGP62221, 103.2±13.0%. The LOQ in tissue homogenate was 48 nmol l^−1^ for midostaurin, 82 nmol l^−1^ for CGP 62221, and 357 nmol l^−1^ for CGP52421 e2.

#### Potential modulation of multidrug resistance

This was determined using a previously reported method that assessed the capacity of cremophor EL to inhibit MDR^10^. Briefly, the ability of the patient's plasma to modulate *ex vivo* intracellular daunorubicin accumulation in multidrug resistant cells (R100; CCRF CEM cells resistant to 100 ng ml^−1^ vinblastine) was measured using fluorescence detection. The activity of 20 *μ*g ml^−1^ Valspodar (PSC833, an inhibitor of P-glycoprotein) added to the patient's prestudy plasma sample defined 100% reversal (no accumulation of daunorubicin). In addition, 20 *μ*g ml^−1^ midostaurin was added to a prestudy sample for estimation of potential activity, and as an inter-patient control. The modulating ability of day 29 plasma samples was determined relative to this. All assays were performed in triplicate. Blank plasma was spiked with 1.0 *μ*l ml^−1^ cremophor EL and run as an inter-assay control. This concentration of cremophor EL caused approximately 50% inhibition of maximum daunorubicin accumulation (Webster *et al*, 1993). The accuracy of the control was always >75%.

### Sample size

It was planned to recruit 14 patients with disease suitable for biopsy as the first cohort. If no responses were seen, the trial would close. If at least one response occurred, a further 13 patients (not necessarily with disease suited for biopsy) would be recruited. Response and toxicity were assessed using WHO criteria.

## RESULTS

### Patient demographics and prior therapies

Seventeen patients with progressive metastatic melanoma were enrolled in the study (Table 1). All were Caucasian, aged 26–77 (median: 55) years, with 12 males and 5 females. Patients’ median ECOG performance status was 1 at study entry. Twelve had received chemotherapy (most commonly with dacarbazine, cisplatin or carmustine). Six had received immunotherapy: five with *α*-interferon and four with interleukin 2. Seven had also received radiation therapy and six had received tamoxifen.

### Efficacy and safety

No patient obtained a tumour response on midostaurin therapy. Two out of 17 (12%) had stable disease for 50 and 85 days respectively until tumour progression. In one patient (number 12 in Table 1) – the only patient who had not been pretreated for metastatic melanoma – a brief minor tumour response was seen. Fourteen out of 17 (82%) had progressive disease, and one patient was not assessable for response. All patients have died. The median survival was 43 days (range: 8–113 days).

No patient's ECOG performance status improved while on study. Eight (47%) had no change, and nine (53%) patients had worsening of their performance status.

Ten patients discontinued the study due to unsatisfactory therapeutic effect and 7 (41%) patients discontinued from the study owing to nausea, vomiting, diarrhoea and/or fatigue, possibly related to the study drug, in association with lack of therapeutic response. two out of 17 patients died on study, although their deaths were not thought to be related to midostaurin therapy. Two patients required dose modification of midostaurin for nausea or vomiting.

Elevated liver enzymes (alanine transaminase or aspartate transaminase) possibly linked to therapy were recorded in four patients but only one reached grade 2 severity. Four patients had hyperglycemia, one of Grade 2 severity. Anaemia occurred in 12 patients, in five of whom it reached grade 2.

### Biologic activity of midostaurin

All 17 patients had baseline tumour biopsies and plasma samples taken for biologic assays at baseline. Nine patients had repeat biopsies and 12 repeat plasma samples, with the remaining patients discontinuing the study early.

**Figure 2:**
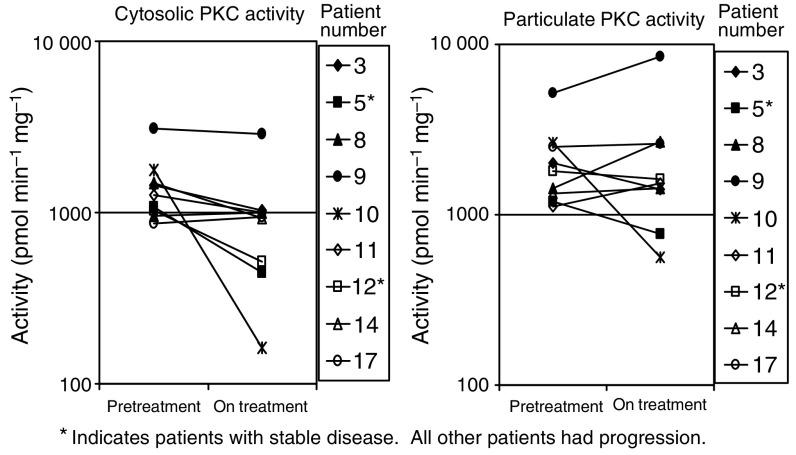
PKC activity in patients’ melanoma biopsies before and during midostaurin therapy.

Results of PKC assay on tumour biopsies are shown in Figure 2. Compared to the pretreatment biopsy, cytosolic PKC activity was reduced by 7–91% in seven out of nine patients. Particulate PKC activity was reduced by 11% to 79% in four out of nine patients. Only one patient (Patient 10) had >50% inhibition in both fractions; however, this patient had progressive disease. On day 0, the percentage of the total PKC activity present in the particulate fraction ranged from 12 to 37% and on day 28 it ranged from 11 to 35%. The percentage of the total PKC activity present in the particulate fraction fell from day 0 to day 28 in two out of nine patients – in patient 10 from 19.1 to 15.4% and in patient 11 from 15.7 to 11.1%.

**Figure 3:**
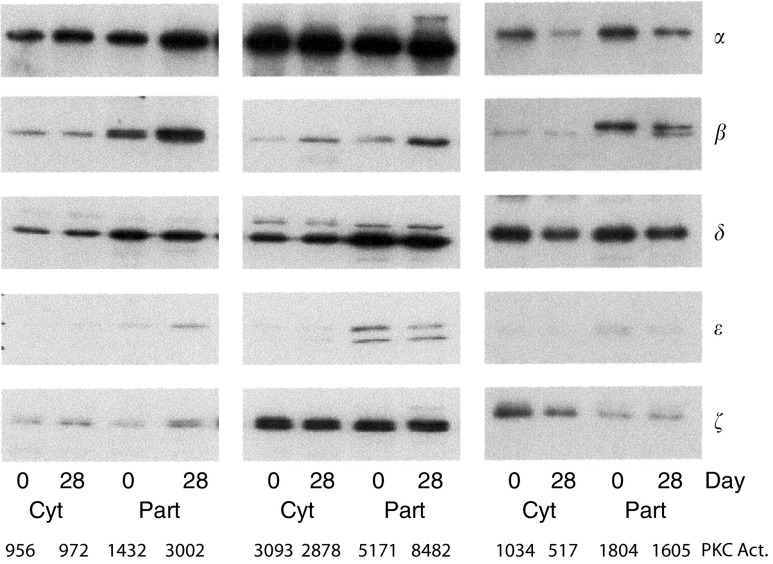
Western blots of PKC isoforms in melanoma biopsies from three patients before and during midostaurin therapy (left to right, patient number 8, 9, 12). Cyt=cytosolic, Part=particulate, PKC Act=PKC activity on corresponding day.

Varying patterns of PKC isoforms were seen in three patients (Figure 3) Protein kinase C alpha was predominant in two patients. However, one of these patients, who was resistant to midostaurin (unchanged cytosolic activity and 65% increase in particulate activity), showed an abundance of PKC*ξ*, an isoform refractory to inhibition by midostaurin (IC_50_>1000 *μ*M). The third patient (patient 12) had predominance of PKC*δ*, which is only moderately sensitive to midostaurin (IC_50_ 0.36 *μ*M). This patient had stable disease for 85 days.

### Modulation of multidrug resistance and tissue/plasma distribution

Addition of 20 *μ*g ml^−1^ midostaurin to pretreatment plasma produced 14–64% (mean 40%) of potential maximal reversal of multidrug resistance. However, plasma from patients receiving midostaurin showed <20% potential maximum reversal in all 12 patients, and in 10 out of 12 patients it was <10%.

### Plasma and tumour concentrations of midostaurin and metabolites

Concentrations of midostaurin and its two metabolites CGP62221 and CGP52421 e2 in plasma and tumour after approximately 1 month 75 mg tid doses are shown in Table 2. In plasma, midostaurin trough concentrations ranged between 771 to 4649 nM (median 2342 nM), and it was ∼2-fold lower than CGP62221 (median 5433 nM) and ∼7-fold lower than CGP52421 (18429 nM). In tumour tissue, the concentration differences between midostaurin and its metabolites were less pronounced. Midostaurin tissue concentration 593 nM (median) was comparable to CGP62221 (710 nM), and only ∼2-fold lower than CGP52421 (1516 nM). The tissue to plasma concentration ratio was similarly low between midostaurin and CGP62221, 0.29 and 0.20, respectively, and the ratio for CGP52421 was the lowest, 0.083.

## DISCUSSION

In this phase IIA clinical trial, no activity of midostaurin at the 75 mg tid dosing regimen was observed, and toxicity led to early discontinuation in seven out of 17 (41%) patients with metastatic melanoma. Most patients had received prior chemotherapy, had large metastases amenable to biopsy, and had short life expectancy. However, these results are similar to the negative results of Phase II trial in melanoma with ISIS3521 (an antisense oligonucleotide against PKC*α*) in which no responses were seen in 23 chemo-naïve patients (Fumoleau *et al*, 2000).

Grade 3 toxicity was infrequent, but a constellation of less severe symptoms including nausea, vomiting, diarrhoea and fatigue, contributed to the decision to discontinue midostaurin in seven out of 17 (41%) patients. After the start of this trial, the final report of a Phase I study (Propper *et al*, 2001) suggested 75 mg bid (150 mg day^−1^) as more tolerable midostaurin dosing for long-term administration. However, a recent Phase II trial of midostaurin to inhibit activated Flt3 kinase in acute myeloid leukaemia (AML) found 225 mg day^−1^ well-tolerated and had promising activity (Stone *et al*, 2005).

A major component of this trial was procurement of tumour and plasma samples for evaluation of target inhibition and pharmacodynamic efficacy. Because many patients had disease progression and deteriorating performance status at the first assessment, only nine of the 17 patients were able to have a repeat biopsy performed after 4 weeks of midostaurin treatment. The results of the assay for inhibition of PKC activity were interesting. Despite midostaurin's potency as an inhibitor of the major isoforms of PKC (IC50 22 nM for PKC*α*) (Fabbro *et al*, 2000), it has been a challenge to increase free concentration to an optimal level because of its high plasma protein binding (99%, Novartis internal report). For a plasma concentration of 2342 nM (median), the free concentration will be only 23.4 nM, which is in the same range as the IC_50_ against PKC*α*. Although the two metabolites CGP62221 and CGP52421 are also active against PKC isotypes, their *in vivo* activity is probably limited. CGP62221 has a weaker activity (IC50 120 nM) than that of the parent drug. CGP52421 has a similar activity (IC50 50 nM) as the parent drug and a higher plasma concentration, but its substantially high plasma protein binding (≫99%, Novartis internal report) renders it less active *in vivo* than the parent drug. Thus, considering the large inter-patient variability for midostaurin plasma concentration and the marginal free concentration relative to the IC_50_ value, it was not surprising that the results showed an inconsistent and variable inhibition of PKC activity in melanoma deposits *in vivo*.

In a small sample of three patients’ tumours, two out of three expressed PKC isoforms known to be insensitive to inhibition by midostaurin. However, larger studies on PKC isoforms in human tumours are needed to determine the significance of this finding. Solid tumours such as melanoma may also be poorly perfused and hypoxic, so that low penetration of midostaurin (tissue/plasma ratio median 0.29) into solid tumours may also account for inconsistent intra-tumoral PKC inhibition.

Our results are in contrast to those obtained with midostaurin in AML where inhibition of autophosphorylation of mutated Flt-3 was documented in leukaemic blasts isolated from patients receiving midostaurin (Stone *et al*, 2005). In 70% of AML patients, there was a 50% reduction of peripheral blood blast cells, and in 25% of patients a 50% reduction in bone marrow blast cells.

Because midostaurin had been reported to modulate multidrug resistance, we determined this potential in patients’ plasma. The results showed negligible effect, indicating midostaurin is unlikely to have therapeutic potential as a clinical modulator of such resistance at the dose of 225 mg per day.

Our results show no clinical activity of midostaurin in metastatic melanoma. This schedule did not show pharmacodynamic efficacy against target PKC isoforms. Other trials with midostaurin as a single agent have, however, demonstrated biologic and clinical activity at the same schedule when the patient population was preselected for expression of midostaurin targets, such as mutated FLT3 kinase, in AML patients. Demonstrating efficacy and target modulation in future midostaurin trials may depend on preselecting patients with constitutively activated (phosphorylated) midostaurin targets: PKC isotypes *α*, *β* and *γ*, FLT3, c-kit, KDR, and PDGF receptor *α* and *β*.

## Figures and Tables

**Table 1:** Patient demographics and tumour responses

**Patient**	**Age/sex**	**Prior therapy**	**Days on midostaurin**	**Best response (duration)**
1	52M	Chemotherapy	60	PD
2	51M	Chemotherapy, tamoxifen	15	PD
3	34M	Chemotherapy	58	PD
4	45F	Chemotherapy	16	PD
5	57F	Chemotherapy, tamoxifen, radiation therapy	57	SD (50 days)
6	60F	Chemotherapy, radiation therapy	8	PD
7	52M	Radiation therapy	23	PD
8	68M	Chemotherapy, radiation therapy	33	PD
9	54M	Chemotherapy, tamoxifen, radiation therapy	37	PD
10	53M	Radiation therapy	64	PD
11	63M	Chemotherapy	56	PD
12	74M	None	113	SD (85 days)
13	55M	Chemotherapy, tamoxifen	25	NA
14	59M	Chemotherapy	57	PD
15	77F	Tamoxifen	27	PD
16	26M	Chemotherapy	15	PD
17	56F	Tamoxifen, radiation therapy	57	PD

PD, progressive disease; SD, stable disease (<25% increase overall diameter of any lesion; no new lesions, after at least 4 weeks); NA, not assessable.

**Table 2:** Plasma and tissue concentrations of midostaurin and its metabolites CGP62221 and CGP52421 e2 epimer

		**Plasma concentrations (nM)**			**Tissue concentrations (nM)**			**Tissue/plasma ratios**		
**Subject #**	**Sampling day**	**Midostaurin**	**CGP 62221**	**CGP52421 (e2)**	**Midostaurin**	**CGP 62221**	**CGP52421 (e2)**	**Midostaurin**	**CGP 62221**	**CGP52421 (e2)**
1	28	4649	10132	22448	—	—	—	—	—	—
3	29	1765	2995	13352	1528	1022	1503	0.87	0.34	0.11
7	29	1437	5761	10767	—	—	—	—	—	—
8	29	2297	5280	15869	358	651	776	0.16	0.12	0.05
9	30	3549	5265	20340	698	1143	1644	0.20	0.22	0.08
10	28	3212	5586	20944	4246	2353	3212	1.32	0.42	0.15
11	28	2614	4660	19151	58	175	635	0.02	0.04	0.03
12	29^a^	1639	3227	16633	607	609	1528	0.37	0.19	0.09
13	30^a^	771	2474	11311	266	523	789	0.35	0.21	0.07
14	29^a^	2901	7205	18383	579	759	1031	0.20	0.11	0.06
15	27^a^	2226	5723	19519	4142	2006	2367	1.86	0.35	0.12
17	29^a^	2387	6061	18475	550	661	1569	0.23	0.11	0.08
										
Mean		2454	5364	17266	1303	990	1505	0.56	0.21	0.085
s.d.		1040	2045	3772	1572	685	796	0.60	0.12	0.036
Median	—	2342	5433	18429	593	710	1516	0.29	0.20	0.083
Mimimum	—	771	2474	10767	58	175	635	0.02	0.04	0.033
Maximum	—	4649	10132	22448	4246	2353	3212	1.86	0.42	0.153

a Represent blood sampling times only; tumour tissue samples were collected at 2–7 days before or after blood samples.
